# Editorial Note: Driving rural industry revitalization in the digital economy era: Exploring strategies and pathways in China

**DOI:** 10.1371/journal.pone.0336361

**Published:** 2025-11-10

**Authors:** The PLOS One Editors

The *PLOS One* Editors issue this Editorial Note to inform readers that a work cited in this article [1] as reference #28 was retracted before the article’s publication date. PLOS considers that this reference is not crucial in supporting the research or conclusions reported in [1], but the following sentence is no longer supported:

Section 2.1, paragraph 2, sentence 9: “Scholars posit that the adoption of digital inclusive finance can effectively mitigate the income disparity between urban and rural dwellers.”
